# Dynamic models for ultrasound-switchable fluorescence

**DOI:** 10.1088/1361-6560/ae639f

**Published:** 2026-05-08

**Authors:** Baohong Yuan

**Affiliations:** Ultrasound and Optical Imaging Laboratory, Department of Bioengineering, The University of Texas at Arlington, Arlington, TX 76019, United States of America; Joint Biomedical Engineering Program, The University of Texas at Arlington and The University of Texas Southwestern Medical Center at Dallas, Dallas, TX 75390, United States of America

**Keywords:** ultrasound-switchable fluorescence, dynamic models, sensitivity matrix, deep tissue imaging, high-resolution imaging

## Abstract

*Objective.* Near-infrared fluorescence imaging enables deep-tissue visualization but is limited to millimeter-scale resolution due to photon scattering. Ultrasound-switchable fluorescence (USF) improves resolution by thermally activating fluorophores within a focused ultrasound region. Although experimentally demonstrated, quantitative understanding of the coupled acoustic–thermal–fluorescence–optical processes governing USF signal formation remains limited. This work establishes a dynamic modeling framework to describe USF signal and velocity formation and to explore model-driven strategies for improving imaging performance. *Approach.* A physics-based framework integrating ultrasound pressure and heating, temperature-dependent fluorescence quantum yield, and photon diffusion was developed. Both analytical derivations and numerical simulations were performed to investigate the dynamic behavior of USF signal strength and velocity under different target configurations and optical source–detector geometries. *Main results.* The framework reproduces the dynamic evolution of USF signal strength and velocity. Analytical expressions were derived to estimate separation limits of adjacent targets within the ultrasound focal volume. Velocity-based analysis reveals structural information beyond conventional intensity-based imaging. A time-dependent sensitivity matrix was obtained, indicating improved spatial localization potential for tomographic reconstruction. *Significance.* This study provides a quantitative theoretical basis for dynamic USF imaging and highlights the additional structural information contained in signal velocity. The results suggest that velocity-based analysis may enable differentiation of otherwise indistinguishable features within the focal volume. The derived dynamic sensitivity matrix further supports tomographic reconstruction and may facilitate future deep-tissue super-resolution strategies.

## 1. Introduction

Near-infrared (NIR) fluorescence imaging in biological tissues at centimeter depths has drawn significant attention for disease localization, diagnosis, and treatment guidance (Li *et al* 1996, O’Leary 1996, Ntziachristos *et al* 2000, Ntziachristos and Weissleder 2001, 2002, Anuradha *et al* 2003, Patwardhan *et al* 2005, Zhu *et al* 2013, Okawa and Hoshi 2023, Chekin *et al* 2025). However, because NIR photons are strongly scattered in tissue, spatial resolution is typically limited to several millimeters, which restricts many biomedical applications (Okumura *et al* 2018, Cheng and Wang 2021, Wang and Yao 2024). To overcome this limitation, ultrasound-switchable fluorescence (USF) imaging has been developed in recent years (Yao *et al* 2019, Liu *et al* 2022, Ren *et al* 2023a). USF imaging relies on two primary components: temperature-sensitive fluorescence contrast agents and a focused ultrasound system that locally increases tissue temperature by a few degrees to activate fluorophores in the focal region. Experimental studies have demonstrated that USF can achieve spatial resolution on the order of hundreds of microns at MHz ultrasound frequencies, significantly improving upon conventional diffuse optical imaging.

Beyond imaging resolution improvement, several new phenomena associated with USF have been observed experimentally. For example, focused ultrasound can induce interstitial fluid streaming through ultrasound momentum transfer (SIF-TUM) (Yuan 2022, Ren *et al* 2025), enabling potential control of nanoparticle transport in deep tissue. USF has also been used for temperature imaging through thermally switchable fluorescence behavior (Ren *et al* 2023b, Yao *et al* 2024), and localization-based approaches have been proposed to achieve super-resolution USF imaging (Yu and Yuan 2024). These studies demonstrate the growing experimental capability of USF imaging systems.

Despite these experimental advances, quantitative understanding of USF signal formation remains limited. USF imaging involves strongly coupled acoustic, thermal, fluorophore-response, and photon-transport processes. Experimental measurements alone cannot easily separate the contributions of these mechanisms or predict USF behavior under new imaging configurations. As a result, interpretation of USF signals and optimization of imaging parameters still rely largely on empirical observations. To address this limitation and complement existing experimental studies, a comprehensive dynamic modeling framework for USF imaging was developed to inform and guide future investigations. The model integrates ultrasound pressure and temperature fields, temperature-dependent fluorescence quantum yield, and photon diffusion in tissue into a unified simulation workflow. The goal of this modeling study is to provide a quantitative and physics-based description of USF signal and its velocity formation and to enable exploration of USF behavior under controlled and hypothetical conditions that are difficult to realize experimentally. Using this framework, USF dynamic signals and velocities are simulated and analyzed. A potential capability of using USF signal velocity to differentiate otherwise indistinguishable structures within the ultrasound focal volume is investigated and discussed. A tomographic framework is proposed to potentially enhance resolution in deep tissue. The corresponding sensitivity (weight) matrix is simulated and analyzed to assess the feasibility of USF tomographic reconstruction strategies. These analyses provide insight into USF imaging mechanisms and may facilitate future experimental design and system optimization.

## 2. USF dynamic models

### 2.1. Principle of USF

**Figure 1.**
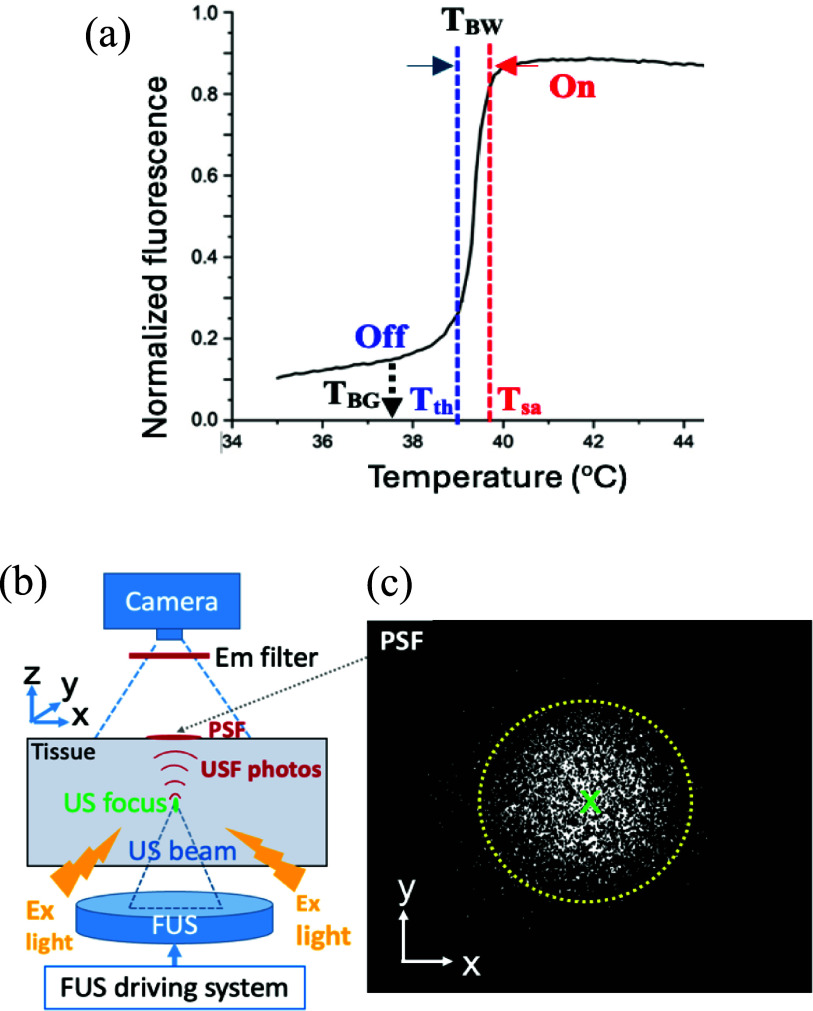
Schematic diagrams showing the USF imaging principle: (a) a step-like function between fluorescence strength and temperature change from a DPPC liposome-based USF nanoagent. The concepts of *T*_th_, *T*_sa_, *T*_BW_, and *T*_BG_ are also indicated in the figure; (b) a setup of USF imaging system and tissue sample; US: ultrasound; FUS: focused ultrasound; Ex: excitation; Em: emission. Note that the excitation light can be illuminated, and the camera can also be positioned at different locations (bottom, top, and/or sides); (c) one example showing USF photon distribution on the tissue surface acquired by the camera and can be called the point spread function (PSF) of the USF imaging system. The yellow circle indicates the contour of the PSF, and the green *x* indicates its center.

The principle of USF imaging is shown in figure 1. Briefly, environment-sensitive NIR fluorophores (such as indocyanine green, ICG) were encapsulated into temperature-sensitive nanoagents (liposomes or nanoparticles) (Liu *et al* 2020, 2022). The fluorescence exhibits a step-like function relative to the environment temperature (figure 1(a)) (Liu *et al* 2022). When the temperature (*T*) is below a threshold (*T* < *T*_th_), they fluoresce weakly (OFF state, figure 1(a)). When *T* is above a saturation threshold (*T*_sa_), they fluoresce strongly with a stable value relative to temperature (ON state, figure 1(a)). When *T*_th_ < *T* < *T*_sa_, the emission (Em) strength sharply increases as temperature rises and is called the transition band (figure 1(a)). The difference between the two temperature thresholds is called transition bandwidth (*T*_BW_ = *T*_sa_–*T*_th_) (Cheng *et al* 2014). The *T*_th_ is also known as LCST (the lower critical solution temperature of the thermo-sensitive nanoagents) (Cheng *et al* 2014). In USF imaging, the threshold *T*_th_ can be controlled slightly above the tissue background temperature (*T*_BG_ = 37 °C) to maintain an OFF state of nanoagents before applying ultrasound. When a focused ultrasound pulse is applied, the tissue temperature at the focus will be increased above the threshold (*T* > *T*_th_) to switch on the fluorophores (figures 1(b) and (c)). The USF nanoagents outside the focus remain off. A USF image can be formed by scanning the ultrasound focus. The spatial resolution is determined by either the acoustic or thermal focal size, whichever is larger (∼0.5–0.8 mm). A 2.5 MHz focused ultrasound transducer (H-108, Sonic Concepts; active diameter 60 mm; focal length 50 mm) was used, with acoustic focal sizes of approximately 0.5 mm laterally and 3.3 mm axially, which is much smaller than the spatial resolution of diffuse optical tomography (DOT) (∼5 mm) (Yao *et al* 2019). In this study, we call this method the conventional USF imaging in which the spatial resolution depends on the ultrasound/thermal focal size. Any structures in the focal volume will not be able to be resolved via this method (i.e. the diffraction limit). Note that the NIR excitation (Ex) light is delivered into centimeters-deep tissue via light scattering, so no need to focus the light beam. The emitted NIR fluorescence photons propagate out of the tissues via light scattering (towards all directions). Therefore, the Ex light source and the camera can be positioned at different locations (bottom, top, and/or sides). As an example, figures 1(b) and (c) respectively shows a diagram of the system setup and a point spread function (PSF) of the USF system on the tissue sample surface (*XY* plane). The ultrasound focus (the green dot in figure 1(b)) is considered a point source to generate the USF photons and propagate out of the tissue. Usually, before ultrasound is applied, the background fluorescence is acquired and subtracted. The difference indicates the USF photons (see the PSF in figure 1(c)). The circle in figure 1(c) indicates the contour of the PSF, and the green *x* indicates the PSF center.

### 2.2. Ultrasound pressure distribution

To calculate the ultrasound-induced temperature increase in the focal volume as a function of space and time, the distribution of the amplitude of the ultrasound pressure wave needs to be calculated. Currently, several numerical software packages are available and can be used for this purpose, such as HIFU simulator (Soneson 2009), Field-II (Jensen and Svendsen 1992), *k*-wave (Treeby and Cox 2010a, 2010b), and so on. However, to improve the simulation speed, in this study, an analytical method (equation (1)) was adopted to simulate the ultrasound pressure amplitude ${P_1}\left( {x,y,z} \right)$, which has been widely adopted in the literature (Sapozhnikov 2012, Prieur and Sapozhnikov 2017, Yuan 2022). In equation (1), ${P_a}$ is a scalar for controlling the spatial peak value of the amplitude ${P_1}\left( {x,y,z} \right)$ at the focal center. ${z_d} = k{\bar a^2}$/2, in which $k = \frac{{2\pi }}{\lambda }$ is the wavenumber ($\lambda $ is the ultrasound pressure wavelength), and $\bar a$ is a factor to control the beam lateral size on the plane *Z* = 0. Like the previous study, $\bar a$ was given a value of 0.5 mm, which is close to the lateral (*X* and *Y*) full-width-at-half-maximum (FWHM) of the focus of the ultrasound transducer that we usually used for USF imaging. The FWHM along the axial direction (*Z*, i.e. the wave propagation direction) of the transducer is 3.5 mm, which usually is longer than the lateral FWHM. The frequency of the ultrasound is 2.5 MHz. Another two factors are ${D_ - } = {x^2} + {y^2} + {\left( {z - {\mathrm{j}}{z_d}} \right)^2}$, ${D_ + } = {x^2} + {y^2} + {\left( {z + {\mathrm{j}}{z_d}} \right)^2}$, and ${\mathrm{j}} = \sqrt { - 1} $.

\begin{equation*}{P_1}\left( {x,y,z} \right) = \frac{{{P_a}{z_d}}}{{2{\mathrm{sin}}{{\mathrm{h}}^2}\left(k{z_d}\right)}}\left[ {{e^{k{z_d}}}\frac{{{\mathrm{sin}}\left( {k\sqrt {{D_ - }} } \right)}}{{\sqrt {{D_ - }} }} - {e^{ - k{z_d}}}\frac{{{\mathrm{sin}}\left( {k\sqrt {{D_ + }} } \right)}}{{\sqrt {{D_ + }} }}} \right].\end{equation*}

The full spatiotemporal expression of the ultrasound pressure can be written as ${P_1}\left( {x,y,z} \right){\mathrm{exp}}\left( { - {\mathrm{j}}\omega t} \right)$, where ${P_1}\left( {x,y,z} \right)$ is the complex spatial amplitude of the ultrasound wave (i.e. equation (1)), and is a function of space ($x,y,z$) and independent of time. The time-dependent term ${\mathrm{exp}}\left( { - {\mathrm{j}}\omega t} \right)$ represents the harmonic oscillation of the pressure field at angular frequency $\omega\;\left( { = 2\pi f} \right)$. In this study, only the spatial amplitude is required, and therefore the time-dependent term is omitted (Yuan 2022).

Equation (1) provides an efficient approximation for calculating the acoustic pressure distribution, where the parameter $\bar a$ controls the focal size determined by the transducer geometry. In the USF modeling framework, the thermal field primarily governs fluorescence switching and USF signal formation, and thermal diffusion smooths small-scale acoustic variations within the focal region. Thus, the simplified acoustic model is sufficient for capturing the relevant USF dynamics considered in this study.

### 2.3. Ultrasound-induced temperature distribution

Once the ultrasound field is calculated, the next step is to calculate the dynamic change of the temperature field ($\Delta T\left( {x,y,z,{\text{ }}t} \right)$) induced by tissue absorbing the ultrasound energy (heating) and caused by the thermal diffusion after ultrasound is off (cooling). Like the previous study (Yuan 2022), the bio-heat transfer equation (2) is adopted for this purpose. The difference is that the calculated dynamic temperature distribution will be used to calculate the fluorescence signal strength, rather than investigate nanoparticle transportation via the thermophoresis effect.

\begin{equation*}{\rho _0}{C_t}\frac{{\partial \left( {\Delta T\left( {r,t} \right)} \right)}}{{\partial t}} = {k_t}{\nabla ^2}\Delta T\left( {r,t} \right) - {\omega _b}{\rho _b}{C_b}\Delta T\left( {r,t} \right) + H\left( {r,t} \right).\end{equation*}

Here, the ultrasound-induced temperature rise is defined as ${{\Delta }}T\left( {r,t} \right) = T\left( {r,t} \right) - {T_{{\mathrm{BG}}}}$, where ${T_{{\mathrm{BG}}}}$ denotes the steady-state background temperature in the absence of ultrasound ($H = 0$). Subtracting the steady-state Pennes equation at $T = {T_{{\mathrm{BG}}}}$ from the full equation eliminates the background perfusion and metabolic terms, yielding a governing equation for ${{\Delta }}T$. The perfusion term therefore reduces to a linear decay term $ - {\omega _b}{\rho _b}{c_b}\,{{\Delta }}T$, representing heat removal toward the blood temperature. The specific heat capacity of tissue and blood are represented as ${C_t}$ and ${C_b}$, respectively. Tissue thermal conductivity is denoted as ${k_t}$. Tissue and blood density are indicated as ${\rho _0}$ and ${\rho _b}$, respectively. Blood perfusion rate in tissue is represented as ${\omega _b}$. The term $H\left( {r,t} \right)$ represents the volumetric heat generation rate caused by ultrasound absorption in tissue. Physically, it describes the conversion of acoustic energy into thermal energy as the ultrasound wave propagates through a lossy medium. As the acoustic intensity decays due to absorption, the lost acoustic energy is deposited locally as heat. It can be approximated as $H\left( {r,t} \right) = 2\alpha I\left( r \right)u\left( t \right) = \frac{{\alpha {{\left| {{P_1}\left( {x,y,z} \right)} \right|}^2}}}{{{\rho _0}{c_0}}}\left( {u\left( t \right) - u\left( {t - \Delta t} \right)} \right)$ (Humphrey 2007, Bigelow *et al* 2011). The acoustic intensity $I\left( r \right)$ is related to the acoustic pressure amplitude by $I\left( r \right) = \frac{{{{\left| {{P_1}\left( {x,y,z} \right)} \right|}^2}}}{{2{\rho _0}{c_0}}}$ (Humphrey 2007, Bigelow *et al* 2011). $u$ is a step function so that $\left( {u\left( t \right) - u\left( {t - \Delta t} \right)} \right)$ defines a rectangular function with a pulse width of $\Delta t$. The rectangular function equals 1 during the ultrasound exposure time from 0 to $\Delta t$ and 0 otherwise. Therefore, $\Delta t$ is called ultrasound exposure time. ${\left| {{P_1}\left( {x,y,z} \right)} \right|^2}$ indicates the square of the ultrasound complex amplitude (${P_1}\left( {x,y,z} \right)$, calculated from equation (1). The current work aims to describe the USF dynamics during a single ultrasound exposure at a fixed spatial location. Therefore, pulse repetition frequency is not included in the model, as it primarily affects scanning speed in imaging experiments rather than local USF signal formation. During the exposure interval, the duty cycle is assumed to be 100%. ${c_0}$ is ultrasound speed in tissue. In literature, ultrasound absorption coefficient $\alpha $ is usually expressed as dB m^−1^ MHz^−1^ with a typical value of 58 dB m^−1^ MHz^−1^ or 58/8.686 Np m^−1^ MHz^−1^ for soft tissue (Yuan 2022 and https://itis.swiss/virtual-population/tissue-properties/database/). In this study, $\alpha $ is the ultrasound absorption coefficient after considering the 2.5 MHz frequency effect and therefore has a unit of m^−1^ or Np m^−1^ (see table 1).

**Table 1.** Major variables, their physical meanings and adopted values in simulations.

Variables	Physical Meaning	Unit and value used
*φ_ex_, φ_fl_*	Excitation (ex) or emission (fl) photon fluence rate	(W m^−2^)

$v$	Light velocity	2.9979 $ \times $10^8^ (m s^−1^)

${M_0}$	Light power	0.003 (W) (a constant canceled via normalization)

$\mu _a^{{\mathrm{ex}}},\mu _a^{{\mathrm{fl}}}$	Excitation (ex) or emission (fl) photon absorption coefficient	$\mu _a^{{\mathrm{ex}}} = 15 (\mathrm{m}^{-1}),\;\mu _a^{{\mathrm{fl}}}$ = 12 (m^−1^)

$\mu _s^{\prime{\mathrm{ex}}},\mu _s^{\prime{\text{ }}fl}$	Excitation (ex) or emission (fl) photon reduced scattering coefficient	$\mu _s^{\prime{\mathrm{ex}}}$ =1500 (808 nm), (m^−1^)$\mu _s^{\prime{\text{ }}fl}$ =1700 (830 nm), (m^−1^)

${D_{\textit{ex}}},{\text{ }}{D_{fl}}$	Excitation (ex) or emission (fl) photon diffusion coefficient	$D = v/\left[ {3\left( {{\mu _{\mathrm{a}}} + \mu _{\mathrm{s}}^{\prime}} \right)} \right]$ ${D_{{\mathrm{ex}}}}$ = 4.95 $ \times {10^4}$, (m^2^ s^−1^) ${D_{{\mathrm{ex}}}}$ = 4.38 $ \times {10^4}$, (m^2^ s^−1^)

$\mu _{{\mathrm{eff}}}^{{\mathrm{ex}}}$, $\mu _{{\mathrm{eff}}}^{{\mathrm{fl}}}$	Excitation (ex) or emission (fl) effective attenuation coefficient	${\mu _{{\mathrm{eff}}}} = \sqrt {3{\mu _{\mathrm{a}}}\left( {{\mu _{\mathrm{a}}} + \mu _{\mathrm{s}}^{\prime}} \right)} $, (m^−1^)

${\varepsilon _{{\mathrm{ex}}}},{\varepsilon _{{\mathrm{fl}}}}$	Excitation (ex) or emission (fl) molar extinction coefficient	${\varepsilon _{{\mathrm{ex}}}} = 6.09 \times 10^6$ (808 nm) ${\varepsilon _{{\mathrm{fl}}}} = 6.54 \times {10^5}$ (830 nm) ${{\mathrm{m}}^{ - 1}}{\left( {{\mathrm{mole}}/{\mathrm{liter}}} \right)^{ - 1}}$

$C$	Molar concentration of fluorophore	$4.4 \times {10^{ - 11}}{\text{ }}{\mathrm{mole}}/{\mathrm{liter}}$ (a constant canceled via normalization)

$\alpha $	Tissue ultrasound absorption coefficient. It is a function of frequency, $\alpha {f^y}$. Its unit should be m^−1^ or Np m^−1^ in equation (11) after considering the frequency effect. If it is given as dB m^−1^ MHz^−1^, it should be converted into m^−1^ or Np m^−1^ via the following method, (dB m^−1^ MHz^−1^)/8.686*${f^y}$ → Np m^−1^ or m^−1^. $f$ is the ultrasound frequency in MHz. The power factor is usually $1 \le y \le 2$.	For soft tissue, $\alpha $ is 58 dB m^−1^ MHz^−1^. Frequency = 2.5 MHz and $y = $ 2

${c_0}$	Sound speed in tissue	1540 (m s^−1^)

$\omega $	Angular frequency of the adopted ultrasound	$2\pi \times $ 2.5 MHz

${C_{\mathrm{t}}}$, ${\text{ }}{C_b}$	Specific heat capacity of tissue and blood, respectively	4200, 3780 (J kg^−1^ K^−1^)

${\rho _0}$, ${\rho _{\mathrm{b}}}$	Tissue and blood density, respectively	${\rho _0}$ =1064, ${\rho _b}$ =1060, (kg m^−3^)

${k_t}$	Tissue thermal conductivity	0.6 (W m^−1^ K^−1^)

${\omega _b}$	Blood perfusion rate	0.0189 (s^−1^)

${Q_0}$	Quantum yield at tissue background temperature	0.05

${R_0}$	On-to-off ratio of fluorescence strength	2.6

${R_{{\mathrm{eff}}}}$	Effective reflection coefficient	0.475

${T_{{\mathrm{BW}}}}$	Switching bandwidth of USF nanoagents	0.56 °C

${T_{{\mathrm{BG}}}}$	Tissue background temperature	37 °C

${T_{{\mathrm{th}}}}$	Temperature threshold of the USF nanoagents	38.41 °C

${T_{{\mathrm{sa}}}}$	Saturation temperature of the USF nanoagents	38.97 °C

### 2.4. Fluorescence quantum yield vs temperature

As shown in figure 1(a), USF nanoagents usually exhibit a step-like function between their fluorescence Em and temperature. In this study, to simulate this feature, the quantum yield (and therefore fluorescence Em strength) of the fluorophore is assumed as a step-like function of temperature. To avoid the two impractical 90° corners of a real step function (which is usually not possible in experiments), a complementary error function (erfc) was adopted to construct a normalized function that describes the step-like transition between fluorescence quantum yield and temperature. This choice provides a smooth and continuous approximation of a threshold-like response and yields a Gaussian-shaped derivative, which is convenient for analytical modeling.

\begin{equation*}Q\left( {r,{\text{ }}t} \right) = {Q_0} \cdot \left\{ 1 + \left[ {\left( {{R_0} - 1} \right)/2} \right] \cdot erfc\left[ { - \frac{{\left( {T\left( {r,t} \right) - {T_M}} \right)}}{{\sqrt 2 \sigma }}} \right]\right\} \end{equation*}

\begin{equation*}{T_{\mathrm{M}}} = {T_{\mathrm{th}}} + {T_{\mathrm{BW}}}/2 = {T_{\mathrm{th}}} + \left({T_{\mathrm{sa}}} - {T_{\mathrm{th}}}\right)/2;T\left( {r,t} \right) = {T_{\mathrm{BG}}} + \Delta T\left( {r,{\text{ }}t} \right)\end{equation*}

$Q\left( {r,{\text{ }}t} \right)$ represents the dynamic quantum yield of the USF nanoagent at a location of $r{\text{ }}\left( {or{\text{ }}x,y,z} \right)$ and time $t$. ${Q_0}$ denotes its quantum yield at tissue background temperature ${T_{{\mathrm{BG}}}}$. ${R_0}$ is the on-to-off ratio of fluorescence strength (see the on and off states in figure 1(a)). To make the above equation physically meaningful (i.e. $0 \le Q\left( {x,y,z,{\text{ }}t} \right) \le 1$), ${R_0}$ should be in a range of $0 &lt; {R_0} &lt; 1/{Q_0}$. However, $1 &lt; {R_0} &lt; 1/{Q_0}$ is usually true in practice because the fluorescence strength at the on state is higher than at off state. erfc is the complementary error function. ${T_{\mathrm{M}}}$ is the middle temperature between ${T_{{\mathrm{th}}}}$ and ${T_{{\mathrm{sa}}}}$, and calculated by equation (4). ${T_{{\mathrm{BG}}}}$ is the tissue background temperature and is assumed as 37 °C in this study. $\Delta T\left( {x,y,z,{\text{ }}t} \right)$ is the dynamic temperature distribution induced by ultrasound exposure and calculated from equation (2). The parameter $\sigma $ is related to temperature bandwidth (${T_{\mathrm{BW}}}$) and will be discussed in the section 3.2. Note that variations in fluorescence strength due to microenvironmental factors may exist; however, these variations are typically much smaller than the temperature-induced fluorescence switching that defines the USF signal. In USF imaging, fluorophores are commonly encapsulated in nanoparticles or liposomes, where the internal microenvironment and fluorophore type primarily determine the baseline quantum yield ${Q_0}$. In addition, in USF imaging, the signal is calculated as the fluorescence difference between the ultrasound-on and ultrasound-off states. When the signal is expressed as a relative or normalized change, the influence of ${Q_0}$ variability is further reduced. Consequently, the temperature-dependent quantum-yield model in equation (3) (i.e. $\left[ {\left( {{R_0} - 1} \right)/2} \right] \cdot {\mathrm{erfc}}\left[ { - \frac{{\left( {T\left( {r,t} \right) - {T_{\mathrm{M}}}} \right)}}{{\sqrt 2 \sigma }}} \right]$) captures the dominant fluorescence variation.

### 2.5. Photon diffuse models for Ex and Em light

Because USF images tissue at a depth of centimeters, the photon diffuse model is accurate in simulating the Ex light propagation from a light source (located at the tissue surface) to the ultrasound focus, and the Em light propagation from the focus to a detector (located at the tissue surface). For simplicity, in this study, a semi-infinite geometry setup is adopted, which means only one infinite large plane exists as the boundary between the tissue and air. For such a geometry, an extrapolated zero boundary (EZB) condition can be adopted for calculating the diffused photon distribution on the tissue surface via the mirror image source method (figure 1 shows the schematic diagram of this geometry) (Haskell *et al* 1994, Paasschens and’t Hooft 1998). The real source (*S*) and detector (*D*) are positioned on the physical boundary. When a narrow light beam or a fiber with a small diameter is used to deliver the Ex light into tissue, it can be considered a point source located within the tissue. The depth of this point source can be calculated based on the photon mean free path in the tissue, ${z_{{\mathrm{tr}}}} = 1/\left( {\mu _{\mathrm{s}}^{\prime} + {\mu _{\mathrm{a}}}} \right)$, where $\mu _{\mathrm{s}}^{\prime}$ and ${\mu _{\mathrm{a}}}$ are the reduced scattering coefficient and absorption coefficient of the medium, respectively (Haskell *et al* 1994, Paasschens and’T’thooft 1998). The EZB condition is a simple approximation and widely used in the photon diffuse model. It means that the photon vanishes at a virtual boundary that is separated by a distance ${z_{\mathrm{b}}}$ from the physical boundary. ${z_{\mathrm{b}}} = \frac{{1 + {R_{{\mathrm{eff}}}}}}{{1 - {R_{{\mathrm{eff}}}}}}2D$ in which $D = v/\left[ {3\left( {{\mu _{\mathrm{a}}} + \mu _{\mathrm{s}}^{\prime}} \right)} \right]$ is the photon diffusion coefficient ($v$ is the light speed) in the tissue and ${R_{{\mathrm{eff}}}}$ (0.475 in this study) is the effective reflection coefficient that counts the reflected photons due to the mismatch of the refractive index between the tissue (*n* = 1.333) and air (*n* = 1) (Haskell *et al* 1994, Paasschens and’T’thooft 1998).

#### 2.5.1. Distribution of fluence rate of Ex photons

After considering the boundary condition, the photon fluence rate of the Ex light ${\phi _{{\mathrm{ex}}}}\left( r \right)$ at any location $r$ in the tissue and ${\phi _{{\mathrm{ex}}}}\left( {{r_{\textit{i}}}} \right)$ at any location ${r_{\textit{i}}}$ in the image space (generated by the point source and its image source) can be expressed as (O’Leary 1996, Li 1998)

\begin{equation*}{\phi _{{\textit{ex}}}}\left( {{r_{{{\textit{ss}}}^{\prime}}},r} \right) = \frac{{v{M_0}}}{{4\pi {D_{{\textit{ex}}}}}}{G_{{\textit{ex}}}}\left( {{r_{{{\textit{ss}}}^{\prime}}},r} \right),{G_{{\textit{ex}}}}\left( {{r_{{{\textit{ss}^{\prime}}}}},r} \right) = \left[ {\frac{{{\mathrm{exp}}\left( { - \mu _{{\mathrm{eff}}}^{{\mathrm{ex}}} \cdot \left| {{r_{\textit{s}}} - r} \right|} \right)}}{{\left| {{r_{\textit{s}}} - r} \right|}} - \frac{{{\mathrm{exp}}\left( { - \mu _{{\mathrm{eff}}}^{{\textit{ex}}} \cdot \left| {{r_{{{\textit{s}^{\prime}}}}} - r} \right|} \right)}}{{\left| {{r_{{{\textit{s}^{\prime}}}}} - r} \right|}}} \right],\end{equation*}

\begin{equation*}{\phi _{{\textit{ex}}}}\left( {{r_{ss^{\prime}}},{r_{\textit{i}}}} \right) = \frac{{v{M_0}}}{{4\pi {D_{{\textit{ex}}}}}}{G_{{\textit{ex}}}}\left( {{r_{ss^{\prime}}},{r_{\textit{i}}}} \right),{G_{{\textit{ex}}}}\left( {{r_{ss^{\prime}}},{r_{\textit{i}}}} \right) = \left[ {\frac{{{\mathrm{exp}}\left( { - \mu _{{\mathrm{eff}}}^{{\mathrm{ex}}} \cdot \left| {{r_s} - {r_{\textit{i}}}} \right|} \right)}}{{\left| {{r_s} - {r_{\textit{i}}}} \right|}} - \frac{{{\mathrm{exp}}\left( { - \mu _{{\mathrm{eff}}}^{{\mathrm{ex}}} \cdot \left| {{r_{s^{\prime}}} - {r_{\textit{i}}}} \right|} \right)}}{{\left| {{r_{s^{\prime}}} - {r_{\textit{i}}}} \right|}}} \right].\end{equation*}

Here, the function ${G_{{\mathrm{ex}}}}$ is usually called the propagation function (at the excitation wavelength) derived from the photon diffusion equation and the semi-infinite boundary condition for a point source located at ${r_{\textit{s}}}$. The first and second term in the ${G_{{\mathrm{ex}}}}$ represent the effect from the real and the image source, respectively. All other parameters in the above equations can be considered constants and are explained in table 1.

#### 2.5.2. *Distribution of fluence rate of fluorescence* (*Em*) *photons*

Once the fluence rate of the Ex light at a location of $r$ and its image location ${r_i}{\text{ }}$ are calculated from equations (5) and (6), the fluorescence fluence rate, ${\phi _{fl}}\left( {r,{\text{ }}{r_i},{r_D}} \right)$, generated from these two locations and detected at the location ${r_{\mathrm{D}}}$ can be expressed as follows (O’Leary 1996, Li 1998)

\begin{equation*}{\phi _{{\textit{fl}}}}\left( {{r_{ss^{\prime}}};r;{r_{\textit{i}}};{r_{\textit{D}}};t} \right) = \frac{v}{{4\pi {D_{{\textit{fl}}}}}}{\varepsilon _{{\textit{fl}}}}\left[ {{\phi _{{\textit{ex}}}}\left( {{r_{ss^{\prime}}},r} \right)C\left( r \right)Q\left( {T\left( {r,t} \right)} \right){G_{{\textit{f}}l}}\left( {r,{r_{\textit{D}}}} \right) - {\phi _{{\textit{ex}}}}\left( {{r_{ss^{\prime}}},{r_{\textit{i}}}} \right)C\left( {{r_{\textit{i}}}} \right)Q\left( {T\left( {{r_{\textit{i}}},t} \right)} \right){G_{{\textit{fl}}}}\left( {{r_{\textit{i}}},{r_{\textit{D}}}} \right)} \right]\end{equation*}

\begin{equation*}{G_{{\textit{fl}}}}\left( {r,{r_D}} \right) = \frac{{{\mathrm{exp}}\left( { - \mu _{{\mathrm{eff}}}^{{\mathrm{fl}}} \cdot \left| {r - {r_D}} \right|} \right)}}{{\left| {r - {r_D}} \right|}},{G_{{\textit{fl}}}}\left( {{r_{\textit{i}}},{r_D}} \right) = \frac{{{\mathrm{exp}}\left( { - \mu _{{\mathrm{eff}}}^{{\mathrm{fl}}} \cdot \left| {{r_{\textit{i}}} - {r_D}} \right|} \right)}}{{\left| {{r_{\textit{i}}} - {r_D}} \right|}}.\end{equation*}

Here, ${G_{{\textit{fl}}}}\left( {r,{r_D}} \right)$ and ${G_{{\textit{fl}}}}\left( {{r_{\textit{i}}},{r_D}} \right)$ are the propagation functions of the fluorescence generated at the location $r{\text{ }}$ in the ultrasound focal volume ($F$) and ${r_i}{\text{ }}$ in its image volume ($F^{\prime}$) relative to the EZB, respectively. Similarly, $C\left( r \right)$ and $C\left( {{r_{\textit{i}}}} \right)$ are the fluorophore concentrations at the location of $r$ in $F$ and ${r_{\textit{i}}}$ in $F^{\prime}$, and $Q\left( {T\left( {r,t} \right)} \right)$ and $Q\left( {T\left( {{r_i},t} \right)} \right)$ are the quantum yield functions, determined by the local temperature and the USF agent properties, at the location of $r$ in $F$ and ${r_{\textit{i}}}$ in. Because of the symmetrical features, if $C\left( r \right)$ is known, $C\left( {{r_{\textit{i}}}} \right)$ will be known, which is also true for $Q\left( {T\left( {r,t} \right)} \right)$ and $Q\left( {T\left( {{r_{\textit{i}}},t} \right)} \right)$. All other parameters in the above equations can be considered constants and are explained in table 1.

#### 2.5.3. Final solutions of USF signal and its velocity

The USF signal generated from the volume of $F$ and its image volume in $F^{\prime}$ can be calculated by integrating $r$ and ${r_{\textit{i}}}$ through the two volumes, respectively, which is shown as follows

\begin{equation*}{\phi _{USF}}\left( {{r_{ss^{\prime}}};r;{r_i};{r_D};t} \right) = \iiint {{\phi _{fl}}\left( {{r_{ss^{\prime}}};r;{r_i};{r_D}} \right)}d{\Omega _{FF^{\prime}}}\end{equation*}

\begin{equation*} = \frac{{{v^2}{M_0}{\varepsilon _{fl}}}}{{{{\left( {4\pi } \right)}^2}{D_{ex}}{D_{fl}}}}\left[ {\iiint {{G_{ex}}\left( {{r_{ss^{\prime}}},r} \right)C\left( r \right)Q\left( {T\left( {r,t} \right)} \right){G_{fl}}\left( {r,{r_D}} \right)d{{{\Omega }}_F}} - \iiint {{G_{ex}}\left( {{r_{ss^{\prime}}},{r_i}} \right)C\left( {{r_i}{\text{ }}} \right)Q\left( {T\left( {{r_i},t} \right)} \right){G_{fl}}\left( {{r_i},{r_D}} \right)d{{{\Omega }}_{F^{\prime}}}}} \right]\end{equation*}

$d{{{\Omega }}_F}$ and $d{{{\Omega }}_{F^{\prime}}}$ represent the volume elements in $F$ and $F^{\prime}$, respectively. The spatial integration in equation (9) effectively represents a convolution over the ultrasound focal volume. This spatial averaging is a primary reason why conventional USF signal strength alone cannot resolve fine structures within the focal region, analogous to resolution limits arising from finite Ex volumes in optical microscopy. In addition, equation (9) indicates that the USF signal strength is proportional to the product of the nanoagent concentration and their quantum efficiency, i.e. $C\left( r \right)Q\left( {T\left( {r,t} \right)} \right)$. During a short ultrasound exposure, the nanoagent concentration is not or only minimally affected by the acoustic and thermal fields, whereas the quantum efficiency changes significantly in response to the temperature elevation, thereby generating the USF signal. The quantum efficiency of USF nanoagents, $Q\left( {T\left( {r,t} \right)} \right)$, shows a step function of temperature, which is good for improving signal-to-noise ratio by suppressing background noise and increasing the signal in the focal volume. However, a step function does not help to improve the spatial resolution (see figure 6) while a narrow pulse-like function does (such as a square, Gaussian or delta function) (see figure 7). Fortunately, because the derivative of a step function provides a delta function, investigating the velocity of USF signal becomes highly motivated.

The velocity of the USF signal can be calculated by taking the 1st-order derivative relative to time and expressed below:

\begin{align*}{V_{{\mathrm{USF}}}}\left( {{r_{ss^{\prime}}};r;{r_{\textit{i}}};{r_{\textit{D}}};t} \right) &amp; = \frac{{{\mathrm{d}}{\phi _{{\mathrm{USF}}}}\left( {{r_{ss^{\prime}}};r;{r_{\textit{i}}};{r_{\textit{D}}};t} \right)}}{{{\mathrm{d}}t}}\end{align*}

\begin{align*} &amp; = \frac{{{v^2}{M_0}{\varepsilon _{fl}}}}{{{{\left( {4\pi } \right)}^2}{D_{ex}}{D_{fl}}}}\left[ \iiint {G_{ex}}\left( {{r_{ss^{\prime}}},r} \right)C\left( r \right)\frac{{dQ\left( {r,t} \right)}}{{dT}}\frac{{dT\left( {r,t} \right)}}{{dt}}{G_{fl}}\left( {r,{r_D}} \right)d{\Omega _F} \right. \nonumber\\ &amp; \left. \quad - \iiint {{G_{ex}}\left( {{r_{ss^{\prime}}},{r_i}} \right)C\left( {{r_i}} \right)\frac{{dQ\left( {r_i,t} \right)}}{{dT}}\frac{{dT\left( {r_i,t} \right)}}{{dt}}{G_{fl}}\left( {{r_i},{r_D}} \right)d{\Omega _{F^{\prime}}}} \right]\end{align*}

equation (10) shows that the velocity of USF signal is driven by two major factors: the temperature velocity, i.e. $\frac{{{\mathrm{d}}T}}{{{\mathrm{d}}t}}$, which is determined by equation (2), and the derivative of the quantum yield relative to temperature, i.e. $\frac{{{\mathrm{d}}Q}}{{{\mathrm{d}}T}}$. If the ultrasound exposure time is so short that the effects of the thermal diffusion and the blood perfusion are ignorable, the velocity of the temperature ($\frac{{{\mathrm{d}}T}}{{{\mathrm{d}}t}}$) is proportional to the ultrasound heating rate, $H\left( {r,t} \right)$ (see equation (2) and section 3). On the other hand, the derivative of the quantum yield relative to temperature ($\frac{{{\mathrm{d}}Q}}{{{\mathrm{d}}T}}$) results in a narrow pulse-like function, instead of a step function, and its high nonlinearity provides a potential solution to resolve fine structures in the focal volume, although the spatial convolution is still remained in the equation (10). The details will be discussed in the following section.

## 3. Analytical approximations

Before performing numerical simulations, it is advisable to conduct an analytical examination of the solutions under appropriate approximations, which can provide valuable insight into the USF dynamics.

### 3.1. Thermal confinement

When limiting the ultrasound heating exposure time much shorter than the tissue’s thermal diffusion time constant, the thermal confinement is satisfied so that the thermal diffusion term in equation (2) (the 1st term on the right) can be ignored. Meanwhile, if the blood perfusion-induced heat loss can be ignored (the 2nd term on the right in the equation (2)) during the short ultrasound exposure, the ultrasound-induced temperature rise can be approximated

\begin{equation*}\frac{{\Delta T\left( {r,t} \right)}}{{\Delta t}} \approx \frac{{\alpha {{\left| {{P_1}\left( r \right)} \right|}^2}}}{{{\rho _0}^2{c_0}{C_t}}} = \frac{{2\alpha I\left( r \right)}}{{{\rho _0}{C_t}}} = \frac{{2\alpha {I_{{\mathrm{max}}}}}}{{{\rho _0}{C_t}}}i\left( r \right).\end{equation*}

The relationship between ultrasound intensity ($I\left( r \right)$) and amplitude (${P_1}\left( {x,y,z} \right)$) is expressed as, $I\left( r \right) = \frac{{{{\left| {{P_1}\left( {x,y,z} \right)} \right|}^2}}}{{2{\rho _0}{c_0}}}$ (Humphrey 2007, Bigelow *et al* 2011), and used in equation (11). Also, $I\left( r \right)$ is assumed to be denoted as ${I_{{\mathrm{max}}}}i\left( r \right)$, in which ${I_{{\mathrm{max}}}}$ is the maximum acoustic intensity and $i\left( r \right)$ represents the normalized acoustic spatial distribution. Thus, the temperature velocity in equation (10) can be represented as

\begin{equation*}\frac{{{\mathrm{d}}T\left( {r,t} \right)}}{{{\mathrm{d}}t}} = \frac{{{\mathrm{d}}\left( {{T_{{\mathrm{BG}}}} + \Delta T\left( {r,t} \right)} \right)}}{{{\mathrm{d}}t}} \approx \frac{{2\alpha {I_{{\mathrm{max}}}}}}{{{\rho _0}{C_t}}}i\left( r \right).\end{equation*}

This result suggests that, under thermal confinement, the heating rate can be approximated as a time-independent but spatially varying function, with its maximum value linearly proportional to $\alpha $ and ${I_{{\mathrm{max}}}}$. On the other hand, the temperature change is linearly proportional to the ultrasound exposure time ($\Delta t$). Based on our previous experience in *ex vivo* and *in vivo* USF imaging (Yao *et al* 2019), thermal confinement was satisfied by adopting the ultrasound exposure time ∼0.4 s or shorter. This significantly simplifies the temperature dynamic analysis because its spatial distribution ($i\left( r \right)$) is clearly separated from its temporal change (the ultrasound exposure time, $\Delta t$).

### 3.2. Velocity of quantum yield relative to temperature

For convenience of analytical analysis, the quantum yield of a USF agent has been assumed as an ${\mathrm{erfc}}$ function of the temperature as shown in equation (3) to simulate the experimentally measured step-like relationship between fluorescence intensity and agent temperature. This is mainly because the 1st-order derivative of the ${\mathrm{erfc}}$ function has a format of the Gaussian distribution relative to the temperature, which will make the analyses much easier to understand how the temperature theoretically affects the USF signal and its velocity. Thus, the derivative of the agent’s quantum yield relative to the temperature can be analytically represented as

\begin{equation*}\frac{{dQ}}{{dT}} = {Q_0}\left( {{R_0} - 1} \right)\left\{ {\frac{1}{{\sqrt {2\pi } \sigma }}{\mathrm{exp}}\left[ { - {{\left( {\frac{{\left( {T\left( {r,t} \right) - {T_M}} \right)}}{{\sqrt 2 \sigma }}} \right)}^2}} \right]} \right\}.\end{equation*}

The function in the curly brackets is a normalized Gaussian distribution with an independent variable of temperature $T\left( {r,t} \right)$, a mean of ${T_M}$ and a standard deviation of $\sigma $. The value of the $\sigma $ can be represented by the temperature bandwidth ${T_{{\mathrm{BW}}}}$ (i.e. $\sigma = {T_{{\mathrm{BW}}}}/\left( {2\sqrt {2{\mathrm{ln}}\left( 2 \right)} } \right)$) if the ${T_{{\mathrm{BW}}}}$ is defined as the FWHM of the Gaussian distribution. On the other hand, because ${T_{{\mathrm{BW}}}}$ is defined as ${T_{{\mathrm{sa}}}} - {T_{{\mathrm{th}}}}$ as shown in equation (4), the ${T_{{\mathrm{th}}}}$ and ${T_{{\mathrm{sa}}}}$ respectively correspond to the left and right temperature points at which the FWHMs are achieved on the Gaussian curve. In practice, one can measure the agent’s fluorescence Em strength as a function of temperature. Based on these data, one can get the step-like curve and its 1st-order derivative curve relative to temperature. Based on the derivative curve, one can find the FWHM and its corresponding left and right temperature values that can be defined as the ${T_{{\mathrm{th}}}}$ and ${T_{{\mathrm{sa}}}}$, and then the ${T_{{\mathrm{BW}}}}$ and $\sigma $ can be calculated.

Note that the complementary error function in equation (3) and the Gaussian function in equation (13) are mathematically related to a cumulative distribution function (CDF) and its corresponding probability density function (PDF), respectively. Such CDF-PDF structures are commonly encountered in many areas of applied mathematics and physics. For example, in diffusion and heat-transfer theory, error-function solutions describe smooth concentration or temperature transitions, while their derivatives represent localized gradients or flux responses. Similar cumulative-density relationships also appear in other quantitative disciplines, such as financial mathematics, where sensitivity measures (e.g. Delta and Gamma in the Black–Scholes framework) exhibit CDF-like and PDF-like profiles. Although the underlying mechanisms differ across fields, the shared mathematical structure illustrates that using smooth transition functions and their derivatives is a well-established analytical strategy. While the actual fluorophore response may not strictly follow an ideal complementary error function, this formulation captures the essential switching behavior and enables tractable theoretical analysis of USF signal dynamics.

### 3.3. USF signal and its velocity

Inserting equation (11) into equation (4), the resultant into equation (3), and then into equation (9), one can get an analytical expression for the USF signal (not displayed to avoid redundant presentation). By inserting equations (12) and (13) into equation (10), the velocity of USF can be further represented as

\begin{align*} {V_{USF}}\left( {{r_{ss^{\prime}}};r;{r_i};{r_D};t} \right) &amp; = \frac{{{v^2}{M_0}{\varepsilon _{fl}}}}{{{{\left( {4\pi } \right)}^2}{D_{ex}}{D_{fl}}}}\left( {\frac{{{Q_0}\left( {{R_0} - 1} \right)}}{{\sqrt {2\pi} \sigma }}} \right)\left( {\frac{{2\alpha }}{{{\rho _0}{C_t}}}} \right) \nonumber\\ &amp; \quad \times \left[ \iiint {{G_{ex}}\left( {{r_{ss^{\prime}}},r} \right)C\left( r \right)\exp \left[ { - {{\left( {\frac{{\left( {T\left( {r,t} \right) - {T_M}} \right)}}{{\sqrt {2}\sigma }}} \right)}^2}} \right]I\left( r \right){G_{fl}}\left( {r,{r_D}} \right)d{\Omega _F} - }\right. \nonumber\\ &amp;\left. \quad \iiint {{G_{ex}}\left( {{r_{ss^{\prime}}},{r_i}} \right)C\left( {{r_i}} \right)\exp \left[ { - {{\left( {\frac{{\left( {T\left( {r_i,t} \right) - {T_M}} \right)}}{{\sqrt {2}\sigma }}} \right)}^2}} \right]I\left( {{r_i}} \right){G_{fl}}\left( {{r_i},{r_D}} \right)d{\Omega _{F^{\prime}}}} \right] .\end{align*}

Note that all the functions in the second integral (related to ${r_{\textit{i}}}$ in $F^{\prime}$) are mirror image functions relative to the EZB of their corresponding original functions in the first integral (related to $r{\text{ }}$ in $F$). If the original functions are known or can be calculated, these image functions will also be known or calculated. For example, if $C\left( r \right)$, the unknown USF agent concentration distribution, is reconstructed, $C\left( {{r_{\textit{i}}}} \right)$ will be automatically known, which does not increase the unknown number in image reconstruction. Thus, all analyses and discussions can be focused on the first integral without worrying about the 2nd one. equations (14), (9) and (10) tell that the parameters of ${M_0},{\varepsilon _{fl}},{\text{ }}{Q_0},{\text{ }}{R_0},\alpha ,{\text{ }}{\rho _0},{C_t},{D_{ex}}$ and ${D_{{\textit{fl}}}}$ can affect the absolute values of the USF signal and its velocity. However, in practice normalized USF signal or velocity may be used to avoid their effects because these parameters are usually either not accurate or fluctuated or unknown. ${\mu _a},{\text{ }}\mu _s^{\prime}$ and $\mu _{{\mathrm{eff}}}^{{\mathrm{ex}}}$ are included in ${G_{{\mathrm{ex}}}}$ and ${G_{{\mathrm{fl}}}}$ and cannot be canceled via the normalization method. $C\left( r \right),$ $T\left( {r,t} \right),{\text{ }}$ ${T_M}$, $\sigma {\text{ }}({\mathrm{i}}.{\mathrm{e}}.,{\text{ }}{T_{BW}})$, and $I\left( r \right)$ (or $i\left( r \right)$) are in the integral so that they cannot be canceled and should be known or to be reconstructed. Note that these analytical analyses are only for helping to understand the USF mechanism, related phenomena, and numerical simulation results (more analyses will be given in the following discussion sections). The numerical simulation in the next section will be based on the original equations (1)–(10), rather than equations (11)–(14).

Note that when only a single voxel within the ultrasound focal volume contains nanoagents, the spatial integrations in equation (14) reduce to a single-point contribution, leading to a PSF of the USF signal velocity on the tissue surface. In this case, the temporal profile of the PSF exhibits a Gaussian-like (narrow pulse-like) shape. This arises from the exponential dependence of the quantum-yield derivative and the approximation that the local temperature $T\left( {r,t} \right)$ increases approximately linearly during the ultrasound exposure interval. The resulting pulse width is governed by the temperature transition bandwidth of the nanoagents (T*_BW_*) and the local heating rate, which is determined by the ultrasound intensity $I\left( r \right)$. A quantitative expression describing this relationship is provided in figure S2 (supplementary information), which further illustrates how such a narrow temporal pulse can potentially enhance the resolution of fine structures within the focal volume.

## 4. Numerical simulations

### 4.1. Simulation methods

Ultrasound is a mechanical wave and controlled by a wave equation. The ultrasound wavelength adopted in this study is 616 $\mu \mathrm{m}$ (calculated by via the ultrasound velocity of 1540 m s^−1^ and the frequency of 2.5 MHz). Therefore, a small voxel size (12.5 $ \times $ 12.5$ \times $ 43.75 $\mu {m^3}$) was adopted for calculating ultrasound pressure using equation (1). The ultrasound-induced temperature distribution (equation (2)) will be numerically solved using a conventional finite difference method with a zero initial condition and a zero-gradient boundary condition (Yuan 2022). A relatively large voxel size (50 × 50 × 175 $\mu \mathrm{m}^{3}$) was adopted because temperature distribution is determined by the thermal diffusion equation, which does not need a small voxel size. A Cartesian coordinate system was adopted with the origin located at the ultrasound focal center. Like the previous study (Yuan 2022), a zero-gradient boundary condition was adopted for thermal simulation (i.e. $\frac{{\partial \Delta T}}{{\partial l}} = 0$, where $l$ can be *x, y*, or *z*) because the tissue is much larger than the ultrasound focus. The initial condition was $\Delta T\left( {r,t = 0} \right) = 0$. Because equations (9) and (10) involve three-dimensional spatial integration, their computation takes considerably longer than that of the other equations.

The calculation steps are summarized below. (1) The equation (1) is a steady-state function and only involves spatial calculation. Once it is solved, the results of ${P_1}\left( {x,y,z} \right)$ will be input into Equation (2). (2) Because equation (2) involves both space and time, the results of $\Delta T\left( {r,{\text{ }}t} \right)$ will be a spatial and temporal function and will be given to equation (3). (3) The temperature change $\Delta T\left( {r,{\text{ }}t} \right)$ can be converted into the quantum yield change $Q\left( {r,{\text{ }}t} \right)$ (and $Q\left( {{r_i},{\text{ }}t} \right))$ by calculating equations (3) and (4), which is also a spatial and temporal function. 4) Inserting $Q\left( {r,{\text{ }}t} \right)$ (and $Q\left( {{r_i},{\text{ }}t} \right))$ into equation (9), the USF signal can be calculated. 5) Making the 1st-order derivative of the USF signal calculated from the step 4 relative to time can get the velocity of USF signal. 6) To calculate the sensitivity matrices of the USF signal and its velocity, only a single voxel is filled with USF nanoagents. The location of this voxel is scanned through the entire calculation volume. At each scanning location, the USF signal and its velocity are calculated for a light source-detector pair. Eventually, the normalized USF signal and the normalized velocity at each specific scanning location are used to represent the sensitivity at that position. Thus, the sensitivity matrices of the USF signal and its velocity can be formed.

### 4.2. Simulated results for pressure, temperature, and quantum yield

**Figure 2.**
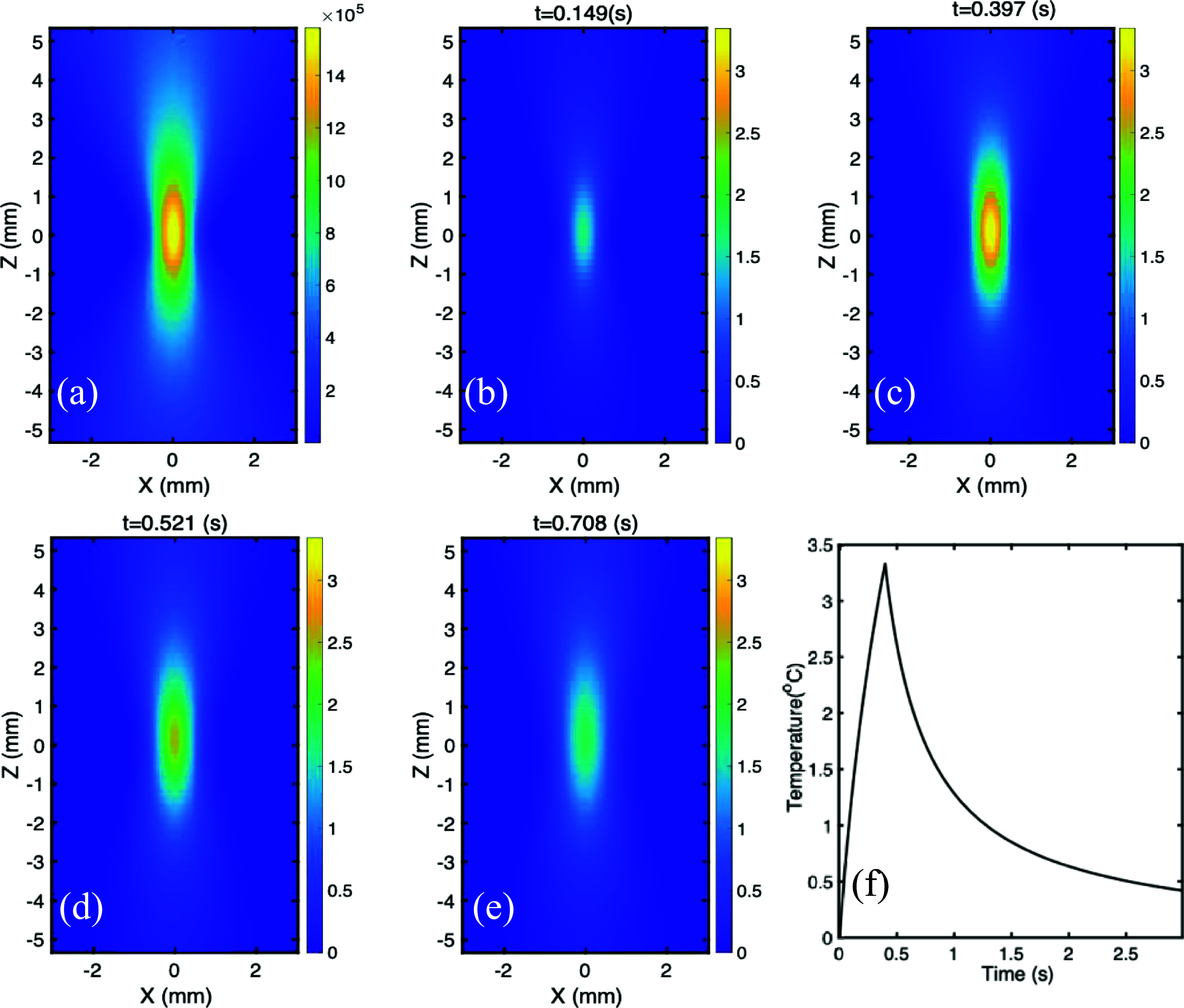
(a) Simulated distribution of the pressure amplitude (*P*_1_) on the *XZ* plane; (b)–(e) simulated temperature dynamic variation on the *XZ* plane at different time points. The ultrasound was exposed for 0.4 s so that (b)–(c) and (d)–(e) represent the heating and cooling period, respectively. (f) The temperature dynamic change as a function of time at the focal center.

Table 1 shows all the parameters used in the simulation (Curra *et al* 2000, Jacques 2013, Yuan 2022). The distributions of the ultrasound pressure amplitude (i.e. amplitude of ${P_1}$) and the ultrasound-induced temperature at different times are displayed in figure 2. The peak pressure amplitude at the focal center is about 1.58 MPa (figure 2(a)) while the ${P_a}$ in equation (1) is 2.4 MPa. This is because ultrasound is attenuated by the medium during the propagation. The FWHMs along the lateral (*X*) and axial (*Z*) directions are 0.855 and 4.495 mm, respectively, which is controlled by the parameter of $\bar a$ in equation (1). The lateral FWHM (0.855 mm) is slightly larger than the value of $\bar a$ (0.5 mm). This indicates that $\bar a$ controls these FWHMs of the simulated ultrasound pressure distribution but is not exactly equal to the lateral FWHM.

Figures 2(b)–(e) shows the temperature change ($\Delta T$) distribution on the *XZ* plane at different times during ((b–c), heating period) and after ((d–e), cooling period) ultrasound exposure. The tissue is exposed with ultrasound from 0 to 0.4 s. The peak central focal temperature in figure 2(c) is 3.34 °C, which is within the range of our experimentally adopted value to switch on USF nanoagents. The lateral (*X*) FWHMs of thermal focus in figures 2(b)–(e) are 0.681, 0.766, 0.891, and 1.047 mm, respectively, while the axial (*Z*) ones are 2.92, 3.317, 3.877 and 4.593 mm, respectively. Compared with the pressure’s FWHMs (figure 2(a)), a conclusion can be drawn that, in the current setup, the thermal lateral and axial FWHMs gradually increase during the ultrasound exposures and are smaller than the corresponding acoustical ones. This is mainly due to two reasons. First, the thermal focus is formed after the ultrasound exposure and accumulated during the exposure. Second, the ultrasound exposure time is so short that the thermal confinement is satisfied, thus the thermal diffusion is minimized (or ignorable). After the exposure, the thermal focal FWHMs are still growing due to the thermal diffusion, which may grow larger than the corresponding acoustical ones. In addition, the value of $\Delta T$ quickly decays during the cooling period. Figure 2(f) shows the peak temperature dynamic change ($\Delta T$) at the focal center as a function of time. The temperature rise during the exposure period almost follows a straight line (except for the late stage of the exposure period). This result validates the thermal confinement and the prediction of the linear relationship between $\Delta T$ and $\Delta t$ indicated in equation (11). The decay of the temperature at the beginning of the cooling period is fast but becomes slow during the late stage of the cooling period.

During the short ultrasound exposure considered in this study (typically Δ*t* ⩽ 0.4 s), the temperature dynamics are dominated by the direct ultrasound heating term, while thermal diffusion and blood perfusion play secondary roles within the exposure window. The thermal diffusivity is defined as ${\alpha _{{\mathrm{th}}}} = \frac{{{k_t}}}{{{\rho _0}{C_t}}},$ and reported values for soft biological tissues are on the order of $\left( {1.2{\mathrm{-}}1.6} \right) \times {10^{ - 7}}{\text{ }}{{\mathrm{m}}^2}/{\mathrm{s}}$ (Anand and Kaczkowski 2008). Using the characteristic thermal diffusion length for transient conduction, ${L_{\mathrm{d}}} \approx \sqrt {4\,{\alpha _{{\mathrm{th}}}}\,{{\Delta }}t} ,$ the diffusion length for Δ*t* = 0.4 s is ${L_{\mathrm{d}}} \approx 0.45$ mm, which is smaller than the ultrasound focal dimensions used here; therefore thermal confinement is maintained and diffusion does not dominate the short-time heating trend. Similarly, the characteristic perfusion time constant in the Pennes model can be written as $\tau = \frac{{{\rho _0}{C_t}}}{{{\omega _b}{\rho _b}{C_b}}}{\text{ }}$. The perfusion-related attenuation during the ultrasound exposure is approximately $1 - {\mathrm{exp}}\left( { - {{\Delta }}t/\tau } \right) \approx {{\Delta }}t/{\tau _p}$ when ${{\Delta }}t \ll \tau $. Representative perfusion rates in soft tissues are typically on the order of 10^−3^–10^−2^s^−1^, with higher values reported in highly perfused organs such as kidney (Pennes 1948, Alkhwaji *et al* 2012). The adopted parameter values in this study fall within these reported ranges, yielding a perfusion time constant of approximately *τ* ≈ 59 s. For a 0.4 s exposure, the perfusion-induced attenuation is approximately 0.7%, indicating that the influence of blood perfusion on short-time temperature dynamics is minimal. Consequently, while the proposed framework can readily accommodate different tissue parameters by substituting ${k_t}$ and ${\omega _b}$, the main conclusions regarding USF short-time signal and velocity formation are expected to be robust across common soft tissues under thermal-confinement operation.

**Figure 3.**
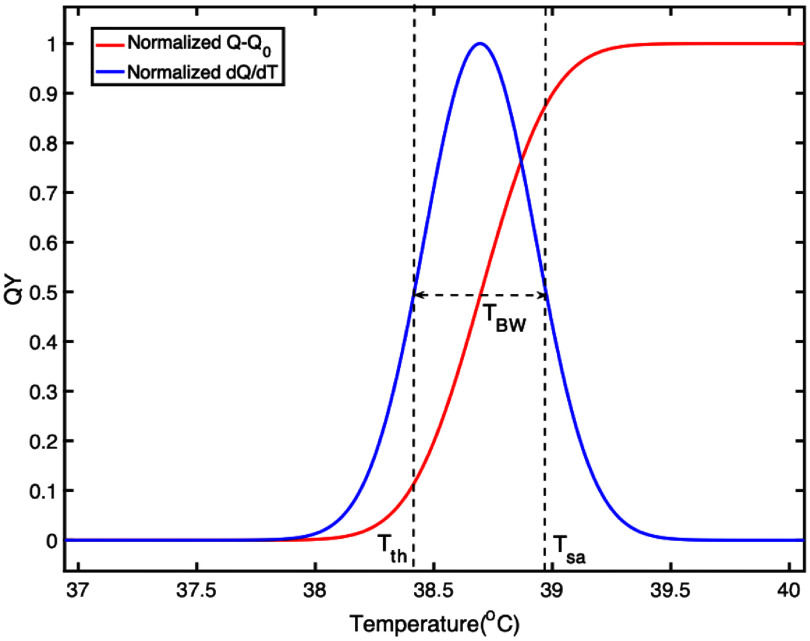
Quantum yield (*Q*–*Q*_0_) as a function of temperature (red) and its first-order derivative (d*Q*/d*T*, blue), and the concepts of defining *T*_th_, *T*_sa_ and *T*_BW_.

Figure 3 shows the results of the quantum yield ($Q$) as a function of temperature and its normalized 1st-order derivative relative to temperature (d*Q*/d*T*) calculated from equation (3). To display two curves in one figure, ${Q_0}$ (0.05) was subtracted from $Q$ and then normalized. The on-to-off ratio of ${R_0}$ was 2.6, which is a typical value for our USF nanoagents. $\sigma $ was given a value of $1/\left( {3\sqrt 2 } \right)$. Thus, based on the definition of ${T_{{\mathrm{BW}}}}$ and its relationship with $\sigma $, ${T_{{\mathrm{BW}}}}$ was calculated as 0.56 °C (i.e.2${\sqrt{\mathrm{ln}(2)}/3}$)), which is equal to the FWHM of d*Q*/d*T* (0.56 °C). Based on the FWHM of d*Q*/d*T*, the values of and ${T_{{\mathrm{sa}}}}$ can be found as 38.41 and 38.97 °C, respectively, and therefore the ${T_M}$ is found 38.69 ^o^C and is close to the value (38.7 °C) given in equation (3) before the original calculation of *Q*. The ignorable 0.01 °C is due to the numerical calculation error. These agreements are not surprising because equation (3) is adopted on purpose due to its 1st-order derivative is a Gaussian function (i.e. equation (13)) and an exact relationship between the $\sigma $ and the FWHM of a Gaussian function exists. It is obvious that the definition of ${T_{{\mathrm{BW}}}}$ via the FWHM is arbitrary and other definitions can also be adopted, such as adding a scaling factor on the FWHM or other non-FWHM methods. However, it will be very helpful to understand the temporal-and-spatial variations of the USF signal and its velocity and analyze their features by simplifying the $Q\left( {r,{\text{ }}t} \right)$ as a complementary error function (and therefore d*Q*/d*T* as a Gaussian function) and defining ${T_{{\mathrm{BW}}}}$ via the FWHM. More discussions will be given in the following sections.

### 4.3. Dynamic USF signal and velocity—(1) a single voxel filled with fluorophores and (2) the entire tissue volume filled with fluorophores

**Figure 4.**
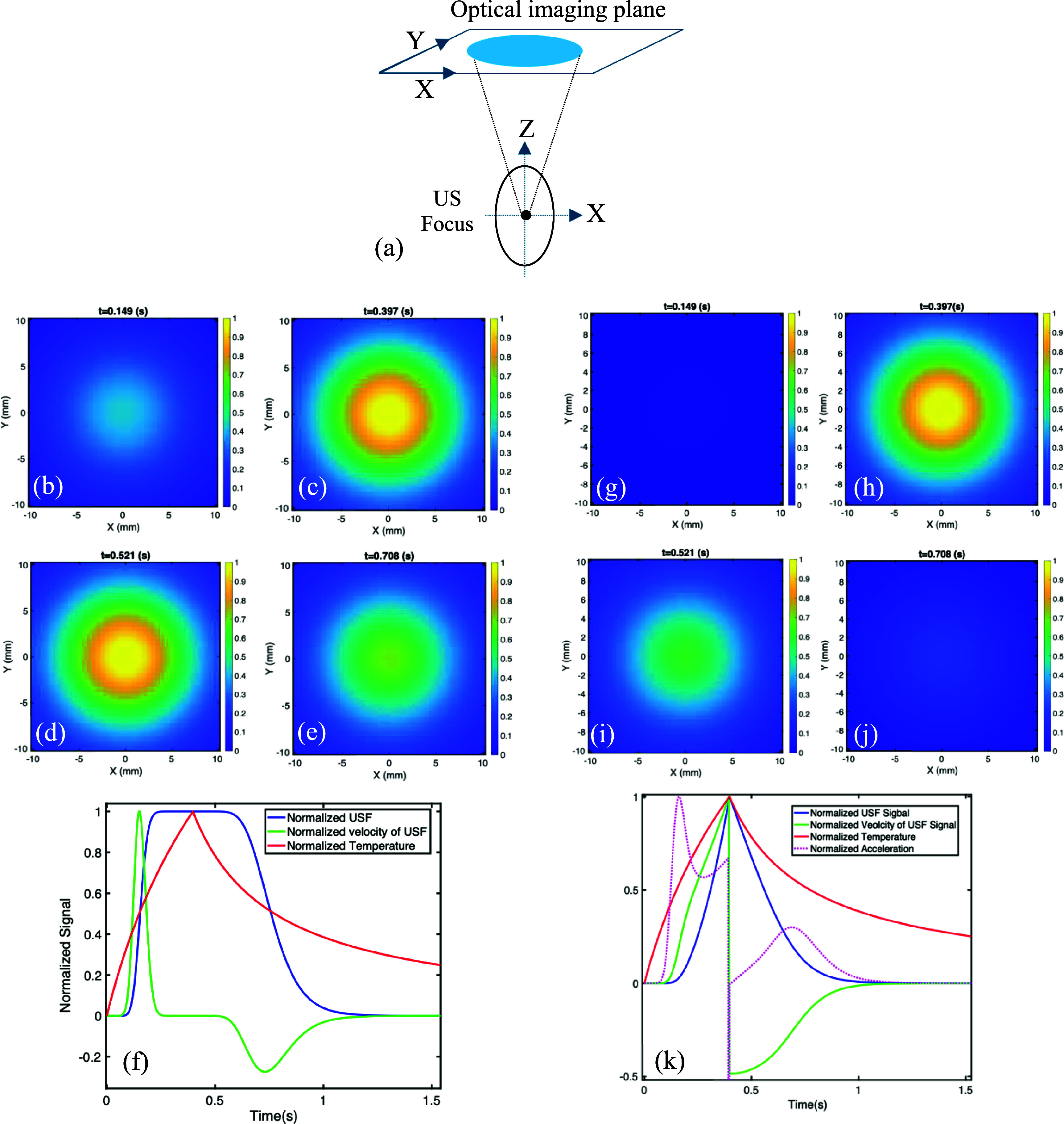
Normalized USF signal and velocity induced by an ultrasound exposure of 0.4 s and a point light source located at the center of the optical imaging plane. (a) A schematic diagram of the simulation geometry. The origin is located at the center of the ultrasound focus. (b)–(e) USF signal 2D distribution on the optical imaging plane at different time (0.149, 0.397, 0.521 and 0.708 s) when only a single voxel at the ultrasound focal center (i.e. the origin) is filled with fluorophores. (f) The dynamic USF signal (blue) and its velocity (green) at the center of the optical imaging plane, and the dynamic temperature change (red) at the ultrasound focal center. (g)–(j) USF signal 2D distribution on the optical imaging plane at different time (0.149, 0.397, 0.521 and 0.708 s) when the entire tissue is filled with fluorophores, and (k) the corresponding USF signal (blue), velocity (green), acceleration (pink), and the dynamic temperature change (red).

**Figure 5.**
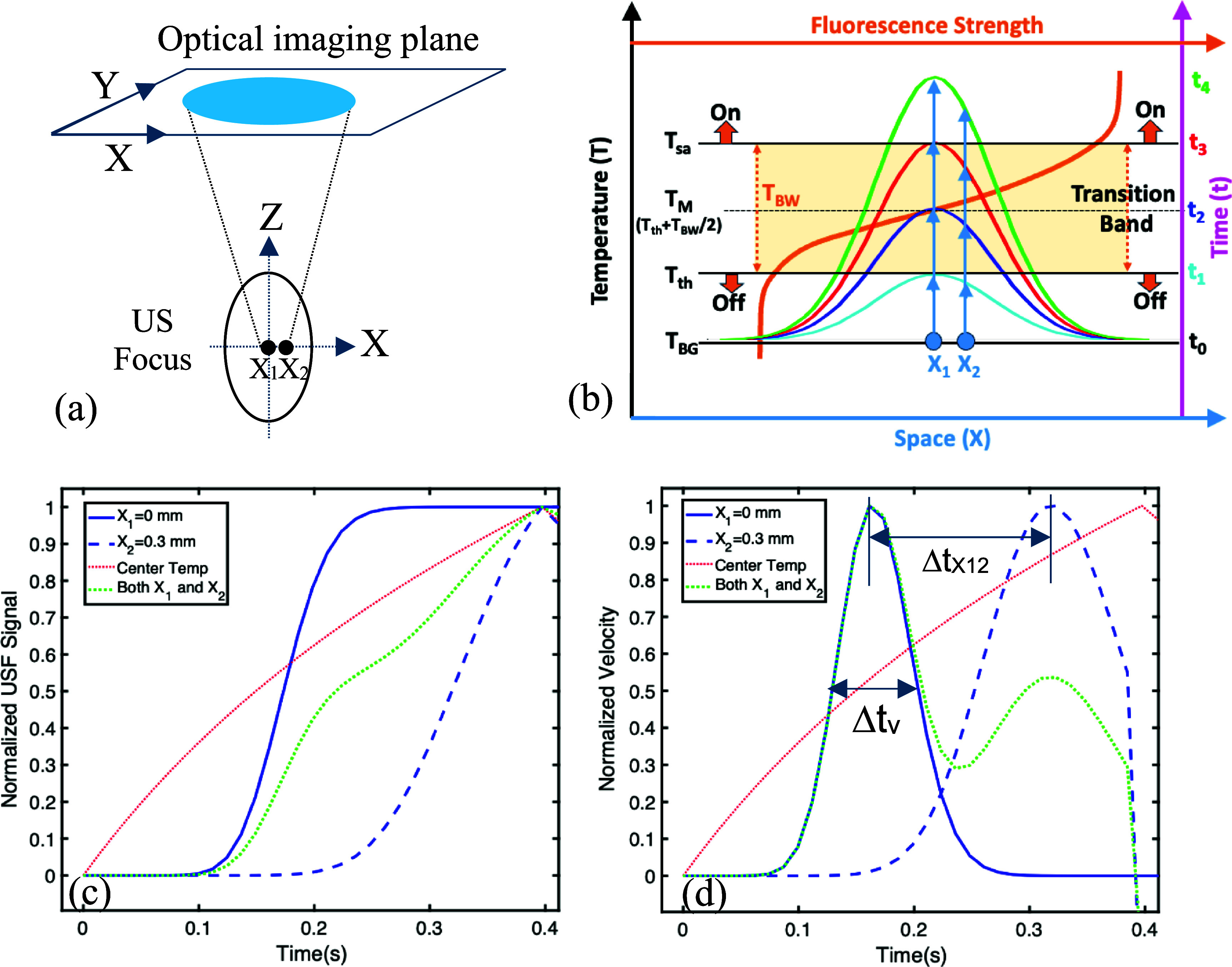
The principle of converting the spatially indistinguishable two point-sources (*X*_1_ and *X*_2_) into a temporal differentiable signal by using the narrow transition band of the USF nanoagent. (a) Configuration of the simulation about the ultrasound focus, optical detection and the two points *X*_1_ and *X*_2_. A point source of the excitation light and a fluorescence detector are located at *x* = *y* = 0 mm and *z* = 10 mm (not shown, on the same side of the tissue and 10 mm above the ultrasound focal center). (b) A schematic diagram to show the principle how the two points *X*_1_ and *X*_2_ are delayed in terms of being switched on and being saturated. (c) Simulated USF signal and (d) its velocity from *X*_1_ and *X*_2_ separated by 0.3 mm in the ultrasound focal volume. (c) Normalized USF signal as a function of time, solid and dashed blue lines are signals from *X*_1_ and *X*_2_; green line from both; red line is the normalized temperature at the *X*_1_. (d) Normalized velocity of USF signal from (c). Δ*t*_v_ is the pulse width of the velocity pulse and Δ*t_X_*_12_ is the time delay between the two velocity pulses generated by *X*_1_ and *X*_2_.

Figure 4(a) displays a schematic diagram of the simulation geometry. The optical imaging plane is on the tissue surface. A point light source was positioned on the tissue surface (i.e. the optical image plane) with coordinates of *x* = *y* = 0 and *z* = 10 mm (not shown on figure 4(a)). The origin of the system was located at the center of the ultrasound focus. Figures 4(b)–(f) and (g)–(k) show the normalized USF signals and velocities for two situations: (1) only a single voxel at the center of the ultrasound focus (i.e. the origin) is filled with USF nanoagents (figures 4(b)–(f)) and (2) the entire tissue is filled with USF nanoagents homogenously (figures 4(g)–(k)). Figures 4(b)–(f) represent the USF signal distribution on the optical imaging plane at 0.149, 0.397, 0.521 and 0.708 s, respectively. The ultrasound exposure time was 0.4 s. Therefore, figures 4(b), (c) and (d), (e) indicate the results during and after the ultrasound exposure, respectively. Clearly, USF photons are widely distributed on the top of the tissue surface because USF photons are generated from deep tissue (10 mm in this example) and propagated to tissue surface via light scattering. figure 4(f) shows the dynamic change of the USF signal (blue) and its velocity (green) at the center of the optical imaging plane, respectively. The red line indicates the ultrasound-induced temperature change at the ultrasound focal center. The USF signal remains ignorable during the initial short period when the temperature is significantly below the temperature threshold (${T_{{\mathrm{th}}}}$). After that, the USF signal sharply increases and reaches a plateau quickly. This is because the temperature rises above the threshold (${T_{{\mathrm{th}}}}$) and quickly goes beyond the saturated temperature (${T_{{\mathrm{sa}}}}$) (also see figure 5(b)). After ultrasound exposure is over, the temperature falls due to the thermal diffusion, and the USF signal remains the plateau level for a certain period and then falls gradually because the temperature falls below the saturated temperature (${T_{{\mathrm{sa}}}}$.). This explains why figures 4(c) and (d) have similar signal strength and spatial distribution (because they are in the signal saturated level). The velocity shows a sharp and positive pulse when the temperature reaches around the middle temperature (${T_{\mathrm{M}}}$) between the ${T_{{\mathrm{th}}}}$ and ${T_{{\mathrm{sa}}}}$ (also see figure 5(b)). The pulse width depends on the temperature bandwidth ${T_{{\mathrm{BW}}}}$ and the heating speed ($\Delta T/\Delta t$, see figure 2 for details). During the cooling period, the velocity shows a negative and relatively broad pulse due to the USF signal slowly falls.

The situation becomes different in figures 4(g)–(k) where the nanoagents are uniformly distributed in the tissue. During the ultrasound exposure period, the USF signal continuously rises and does not reach a saturated level. This is because the heated tissue volume continuously increases during the ultrasound exposure period, which can continuously increase the number of switched-on fluorophores and the USF signal, although the fluorophores in the central focal volume may be saturated. Once the ultrasound exposure is over, the USF signal immediately falls (see the blue line in figure 4(k) and compare figure 4(h) with (i)), which is because the quick temperature falling leads to the reduction of total number of the switched-on fluorophhores. The velocity also continuously rises and falls during the heating and cooling period, respectively. However, the velocity reaches the highest rising rate (i.e. the highest acceleration, see the pink line in figure 4(k)) when the temperature rises around the middle temperature (${T_M}$).

### 4.4. USF dynamic signal and velocity—two separated voxels filled with fluorophores

Figure 5 shows the USF signal and its velocity from two 0.3 mm-separated voxels that were filled with USF nanoagents. Figure 5(a) displays the geometry in which two black dots are in the ultrasound focal volume: *X*_1_ at the center (*x* = *y* = *z* = 0 mm) and *X*_2_ at a location slightly shitted to the right from the center (*x* = 0.3 mm, *y* = *z* = 0 mm), respectively. Obviously, *X*_1_ and *X*_2_ cannot be resolved based on the 2D USF signals on the optical imaging plane because these signals are spatially significantly overlapped due to light scattering. This is understandable because both *X*_1_ and *X*_2_ are in the ultrasound focal volume, which reaches the resolution limit of conventional USF imaging (i.e. the focal size of the ultrasound or thermal beam). However, their USF signal dynamics is very different and can be potentially used to differentiate them.

Figure 5(b) explains the fundamental principle about how USF can potentially resolve the two points, *X*_1_ and *X*_2_, within the ultrasound focus, which cannot be resolved by conventional USF or ultrasound. The dynamic USF signals generated by *X*_1_ and *X*_2_ depend on: (1) the temperature at the two locations (indicated via the Gaussian curves), and (2) the transition bandwidth (${T_{{\mathrm{BW}}}}$) of the USF contrast agents (indicated via the brown shadowed area, and the brown curve representing the switching characteristic curve of the USF contrast agents). If tissue background temperature is *T*_BG_ at *t* = *t*_0_, the temperature at *X*_1_ just reaches the threshold *T*_th_ when *t* = *t*_1_ (see the cyan Gaussian curve). This leads to USF nanoagents starting to be switched on and USF photons being detected. When *t* = *t*_2_, the temperature reaches *T*_M_ at which the fluorescence signal has the highest sensitivity relative to temperature (see the blue Gaussian curve). This leads to the velocity of the USF signal reaching the peak value. In figure 5(b), we illustrated this temperature as the middle of the transition band where the temperature is *T*_M_ = *T*_th_ + *T*_BW_/2. In practice, it can be characterized experimentally and may not be exactly equal to the middle temperature. When *t* = *t*_3_, the temperature reaches the fluorescence saturation threshold *T*_sa_ (see the red Gaussian curve), which leads to the USF signal starting to be saturated and its velocity vanishing. When *t* > *t*_3_ (such as *t* = *t*_4_) the temperature is well above the fluorescence saturation threshold *T*_sa_ (see the green Gaussian curve). Thus, the USF signal from *X*_1_ is saturated and its velocity goes to zero. figures 5(c) and (d) respectively plot the USF signal and its velocity as a function of time generated by *X*_1_ only (solid blue lines), by *X*_2_ only (dashed blue lines), and by both *X*_1_ and *X*_2_ (dotted green lines). Because *X*_2_ has a small distance shifted from *X*_1_ (Δ*X* = 0.3 mm), the acoustic intensity at *X*_2_ is slightly weaker, and its temperature and heating speed are slightly lower than that of *X*_1_ (see the blue upward arrows in figure 5(b)). Thus, the times at which the temperature reaches the three thresholds, *T*_th_, *T*_M,_ and *T*_sa_, will be delayed compared with those at *X*_1_. This can be seen by comparing the dashed blue lines with the solid ones in figures 5(c) and (d). The time delay Δ*t_X_*_12_ indicated in figure 5(d) is caused by the space shit Δ*X* and the differences in temperature and in heating speed (see the quantitative expression of Δ*t_X_*_12_ in figure 2). The smaller Δ*t_X_*_12_ can be resolved the smaller Δ*X* can be differentiated. On the other hand, the velocity of the USF signal exhibits a pulse with a pulse width of Δ*t*_v_ (figure 5(d), also see the quantitative expression in figure 2) that is generated by the transition band (*T*_th_ at *t*_1_ and *T*_sa_ at *t*_3_ in figure 5(b)) and with a peak velocity at *T*_M_ and *t*_2_. When Δ*t*_X12_ ⩽ Δ*t*_v_, *X*_2_ cannot be resolved from *X*_1_. Thus, it sets the spatial resolution limit and can be expressed as $\Delta {\text{X }} = 1/\left\{ {\left| {\nabla I/I} \right| \cdot \left[ {\left( {{T_{\mathrm{th}}} - {T_{\mathrm{BG}}}} \right)/({T_{\mathrm{BW}}}/2) + 1} \right]} \right\}$ (see the derivation in figure 2, and the same equation applies for both *Y* and *Z* directions too and can be denoted as $\Delta Y$ and $\Delta Z$, respectively). It tells that the following factors can increase the resolution: (1) large gradient of the relative acoustic intensity ($\left| {\nabla I/I} \right|$); (2) narrow transition bandwidth (${T_{{\mathrm{BW}}}}$); (3) large temperature difference between the temperature threshold and the tissue background temperature $\left( {{T_{{\mathrm{th}}}} - {T_{{\mathrm{BG}}}}} \right)$. Increasing $\left| {\nabla I/I} \right|$ will increase the difference of the heating rate between the two points (equations (11) and (12)). Increasing $\left( {{T_{{\mathrm{th}}}} - {T_{{\mathrm{BG}}}}} \right)$ will increase the temperature range before switching on a fluorophore (like increasing distance to travel, see figure 3 to understand the effect of the ${T_{{\mathrm{th}}}} - {T_{{\mathrm{BG}}}}$). Both situations will increase the time delay difference between *X*_1_ and *X*_2_ to switch on the fluorophore. This will increase the temporal separation between the two velocity pulses in figure 5(d). Decreasing ${T_{{\mathrm{BW}}}}$ will narrow the pulse width of the two velocity pulses in figure 5(d). All three situations are beneficial to differentiate the two points (i.e. reducing $\Delta X$).

When the ultrasound focus reaches the diffraction limit, shrinking $\left| {\nabla I/I} \right|$ is not possible. Thus, higher resolution can be further generated from the term, $\left( {{T_{{\mathrm{th}}}} - {T_{{\mathrm{BG}}}}} \right)/({T_{{\mathrm{BW}}}}/2)$ > 0, by using narrow ${T_{{\mathrm{BW}}}}$ and positive $\left( {{T_{{\mathrm{th}}}} - {T_{{\mathrm{BG}}}}} \right)$. When $\left( {{T_{{\mathrm{th}}}} - {T_{{\mathrm{BG}}}}} \right)$ = 0, the effect of ${T_{{\mathrm{BW}}}}$ is gone and the higher resolution cannot be achieved, which means the two USF velocity pulses in figure 5(d) will be too close to resolve in the time domain. A typical $({T_{{\mathrm{th}}}} - {T_{{\mathrm{BG}}}})$ is ∼3 °C–4 °C and ${T_{\mathrm{BW}}}$ 0.5 °C–1 °C for liposome USF nanoagents. Therefore, the factor, $\left[ {\left( {{T_{\mathrm{th}}} - {T_{\mathrm{BG}}}} \right)/({T_{\mathrm{BW}}}/2) + 1} \right]$, will be between 7 and 14.3 times to potentially improve the spatial resolution beyond the ultrasound diffraction-limited resolution. The uncertainty in temperature caused by measurement noise, and the uncertainty in acoustic intensity caused by tissue motion will be the negative factors to limit the improvement. It should be noted that these analytical expressions apply to simplified conditions in which only selected voxels contain USF nanoagents and are mainly intended to support theoretical understanding and analyses. In practical imaging scenarios, spatial structures will be much more complicated and the spatial resolution and voxel differentiation rely on tomographic reconstruction using multiple measurements acquired from different angles and scanning positions. To support this direction, a dynamic sensitivity matrix will be introduced in the next section, which describes the dependence of USF signal velocity on spatial location and time.

### 4.5. USF weight (or sensitivity) matrix

**Figure 6.**
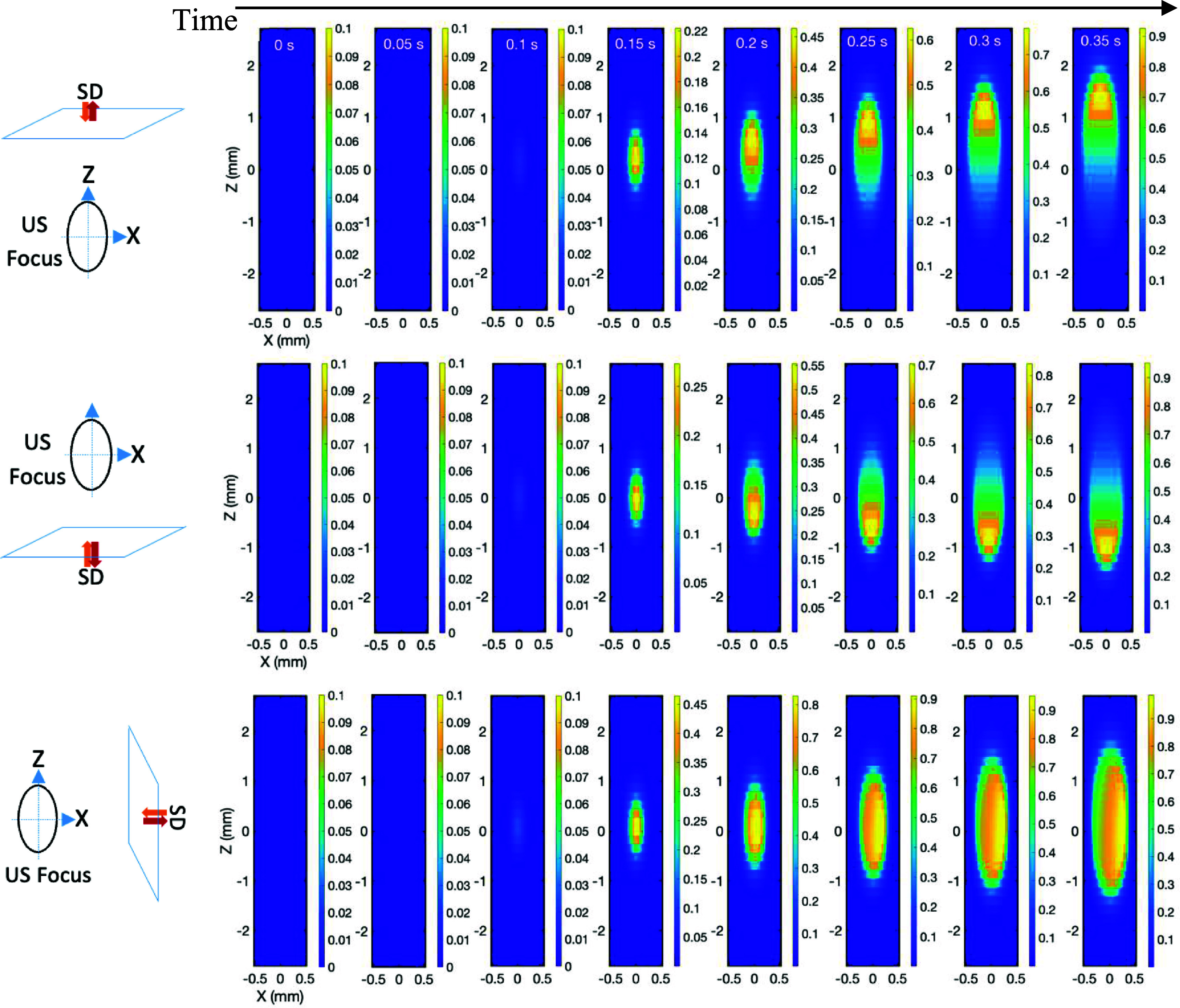
Sensitivity (or weight) distributions of the USF signal at three different configurations of *SD* pairs at different time. The horizontal black line indicates the time from 0 s to 0.35 s with a step size 0.05 s. Each frame shows the sensitivity (or weight) distribution on the *XZ* plane of the ultrasound focus (*Y* = 0 mm). The configuration of the excitation light source (*S*), the detector (*D*) and the ultrasound focus are indicated by three diagrams on the left. The distance between the center of the ultrasound focus and the *SD* plane is 10 mm. The ultrasound wave is always from top to bottom along the *Z* direction.

**Figure 7.**
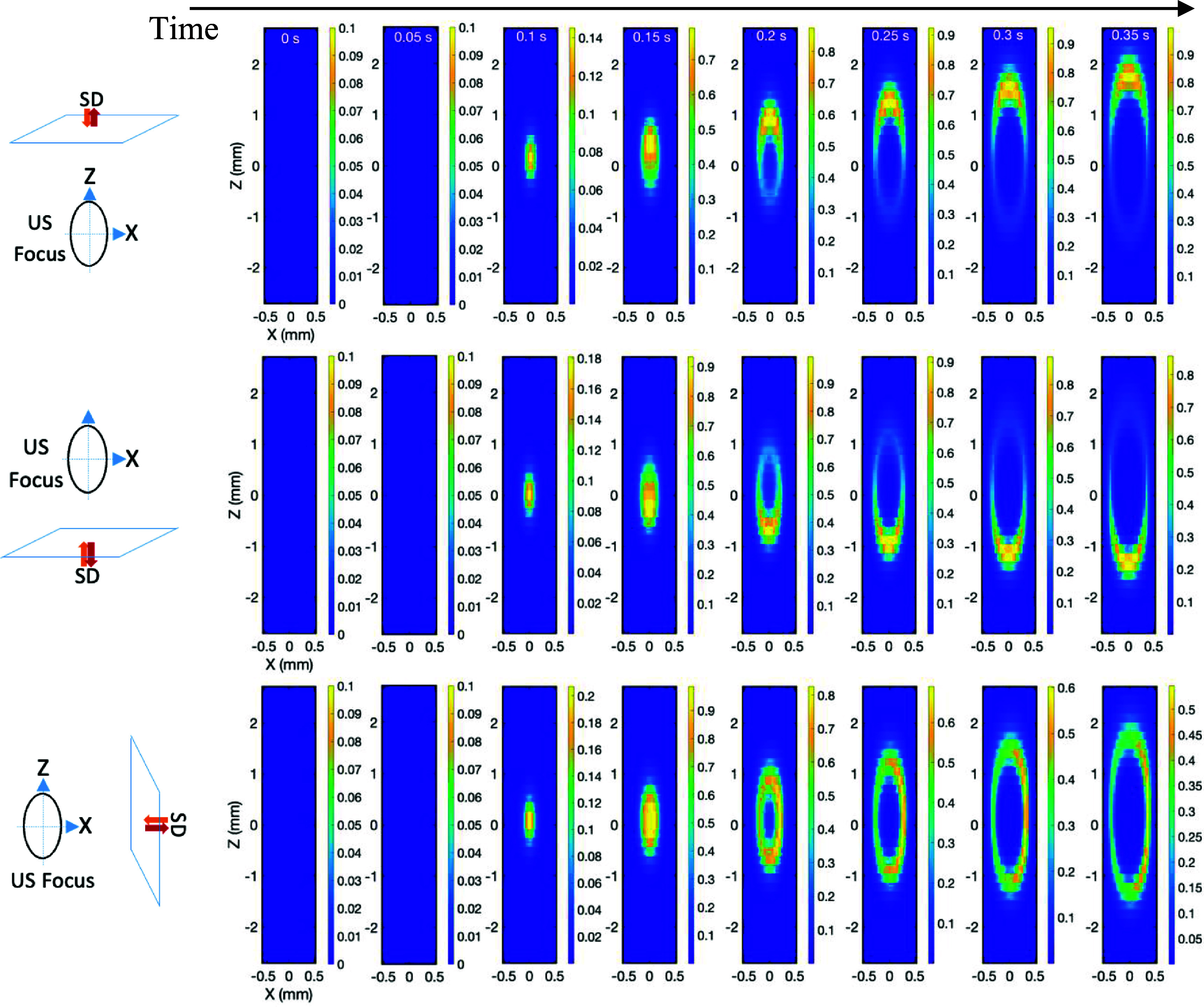
Sensitivity (or weight) distributions of the velocity of USF at three different configurations of *SD* pairs at different time. The horizontal black line indicates the time from 0 s to 0.35 s with a step size 0.05 s. Each frame shows the sensitivity (or weight) distribution on the *XZ* plane of the ultrasound focus (*Y* = 0 mm). The configuration of the excitation light source (*S*), the detector (*D*) and the ultrasound focus are indicated by three diagrams on the left. The distance between the center of the ultrasound focus and the *SD* plane is 10 mm. The ultrasound wave is always from top to bottom along the *Z* direction.

In the tomographic imaging field, a weight distribution is used to describe the contribution weight to the signal from each voxel along the radiation pathway between a source and a detector (O’Leary 1996). In x-ray computed tomography (CT), each voxel has similar weight along the pathway of an x-ray beam. Therefore, these roughly equally weighted voxels cannot be resolved from this single source-detector measurement, and multiple measurements at different angles are needed to differentiate them. Compared with CT, the weight distribution in DOT (O’Leary 1996) is significantly broadened because highly scattered near infrared (NIR) photons are adopted. Also, the weight distribution is not uniform. Voxels close to the source and detector contribute more to the measurement. Thus, it becomes even more difficult to differentiate the voxels in the single source-detector measurement. Mathematically, this increases ill-posedness (Okawa and Hoshi 2023). In USF, the radiation volume is first significantly confined into the ultrasound focal volume. Second, within this volume, the weight distribution is a function of time. As an example, figures 6 and 7 respectively show the weight distributions for USF signal and velocity at different time (from 0 to 0.35 s with a step size 0.05 s during the ultrasound heating period) for three different locations of the light source-detector (*SD*) pairs. Note that the source (*S*) and the detector (*D*) have the same locations in the simulation although they are marked side by side in the figures. The distance between the ultrasound focal center and the *SD* is 10 mm. The results showed that the weight distributions significantly depend on not only the ultrasound-induced thermal dynamic distribution but also on the locations of the *SD* pairs.

In general, the value of the sensitivity of both USF signal (figure 6) and velocity (figure 7) increases as a function of time during the temperature rising period (note that the color bars have different scales). The sensitivity distributions also change. The voxels with high sensitivity (or weight) are significantly constrained in different areas at different times. For example, in the early times (0.1–0.15 s), the high-weighted voxels are located mainly in the central area of the focus (for both USF signal and velocity), which is because the central area has the highest temperature. When temperature continuously increases (0.2–0.35 s), the temperature at the central area rises above the saturation threshold and the fluorescence from this area is saturated. Thus, its contribution to the USF signal strength is saturated (figures 6 and 4), and to the velocity (figures 7 and 4) falls to zero so that the central area becomes dark (i.e. no or low weight). Compared with the USF signal sensitivity, the velocity sensitivity is much sharper in terms of the spatial structure. The sensitivity distribution of the velocity shows a narrow ring structure on the *XZ* plane, meaning that only those voxels in the sensitive area (2D) or volume (3D) will contribute to the velocity significantly while the rest of voxels are insensitive to the velocity. Thus, the number of the sensitive voxels in the velocity matrix (figure 7) is much less than that in USF signal matrix (figure 6). Therefore, we believe that using velocity to image the USF nanoagent distribution in the focal volume is much less ill-posed than using USF signal. This will be studied and reported in future.

Note that the previous analyses in section 3 can be applied for understanding the features in figure 7. The thicknesses of a ring in figure 7 represent voxel numbers with high contribution weights to the USF velocity along different directions at a specific time. It can be analyzed via the equation $\Delta X$ (or $\Delta Y$ or $\Delta Z$) =$1/\left\{ {\left| {\nabla I/I} \right| \cdot \left[ {\left( {{T_{{\mathrm{th}}}} - {T_{{\mathrm{BG}}}}} \right)/({T_{{\mathrm{BW}}}}/2) + 1} \right]} \right\}$ (rather than $\Delta {t_{\mathrm{v}}}$ and $\Delta {t_{X12}}$ in figure 2, which are in time domain). For example, the axial direction (*Z*) shows a thicker ring structure than the lateral direction (*X*). This is because $\left| {\nabla I/I} \right|$ is smaller along the axial direction (*Z*) than lateral direction (*X*), which leads to a larger $\Delta Z$ compared with $\Delta X$. In addition, the ring thickness along different directions are also determined by the factor of $\left[ {\left( {{T_{{\mathrm{th}}}} - {T_{{\mathrm{BG}}}}} \right)/({T_{{\mathrm{BW}}}}/2) + 1} \right]$. Therefore, a larger $\left( {{T_{{\mathrm{th}}}} - {T_{{\mathrm{BG}}}}} \right)$ and/or a narrower ${T_{{\mathrm{BW}}}}$ provide a thinner ring structure, a better resolution, and a less ill-posed inverse problem (figures 2 and S3). Experimental studies need to be conducted in future to validate these predictions.

When the *SD* is located on the top of the ultrasound focus, the contributions from the upper areas of the focus (i.e. shallow tissue) become much more significant compared with those located in the lower area (i.e. deep tissue). This is because the Ex and Em of light from deep tissue are significantly attenuated. In contrast, when moving the *SD* to the bottom or side of the ultrasound focus, the high-weighted voxels also move correspondingly. This is equivalent to using *SD* locations to partially control the weight distribution, which will help to differentiate voxels.

These results indicate that the ill-posedness of the inverse problem can be significantly reduced by providing more constraints via the dynamic change of the sensitivity distribution and multiple measurement in ultrasound and optical setup configurations. For example, changing ultrasound incident angles (such as 0°, ±15°, and ±30° relative to *Z* axis) to conduct multiple measurements at each location of the ultrasound focus. Increasing the spatial sampling density will help to break the elliptical symmetry in the *XZ* and *YZ* planes and the circular symmetry in the *XY* plane of the sensitivity matrix. In addition, more different *SD* pair configurations will also help to break the symmetry and potentially resolve the voxels more accurately (Okawa and Hoshi 2023). While this theoretical framework establishes a foundation for model-based simulation and more comprehensive resolution analysis, further tomographic reconstruction studies using simulated and experimental data should be further studied in future work.

## 5. Conclusions

In this study, we have built a series of models to quantitatively capture the dynamics of USF imaging in deep tissue. Starting from ultrasound pressure distribution, bioheat transfer, quantum yield-temperature step function, and (Ex and Em) photon diffusion, we formulated a unified framework for predicting USF fluence rate and velocity. With these models and well-defined assumptions, analytical solutions were derived to provide clear physical insight into the dynamic behavior of USF. Numerical simulations were further performed to investigate USF signal and velocity dynamics under various fluorescent target configurations (single voxel, two voxels, and tissue uniformly filled with contrast agents). Results reveal that the velocity of USF fluence enables resolution of adjacent targets that remain indistinguishable in conventional USF imaging. Moreover, the sensitivity matrix of the USF velocity was found to be time-dependent and significantly narrower than that of the USF signal itself. This key observation suggests that velocity-based tomographic imaging could surpass the diffraction limit of conventional USF or ultrasound imaging by exploiting the dynamic sensitivity matrix and multiple ultrasound-optical configurations. If implemented successfully, this strategy may pave the way for a new paradigm of USF super-resolution imaging, fundamentally distinct from current super-resolution imaging principles.

## Data Availability

All data that support the findings of this study are included within the article and any supplementary information files. Analytical and Schematic Figures available at https://doi.org/10.1088/1361-6560/ae639f/data1.
